# Determining the Association Between the Origin of Sepsis and the Severity of Sepsis in Intensive Care Unit (ICU) Patients Using Acute Physiology and Chronic Health Evaluation (APACHE) IV

**DOI:** 10.7759/cureus.54653

**Published:** 2024-02-21

**Authors:** Antony J Arumairaj, Imnett Habtes, Hansang Park, Julio C Valencia-Manrique, Jennifer Arzu, Joseph Mattana, Shobhana Chaudhari, Natoushka Trenard, Thomas Newman

**Affiliations:** 1 Internal Medicine, New York Medical College, Metropolitan Hospital Center, New York, USA; 2 Biostatistics, Physician Affiliate Group of New York (PAGNY), New York, USA; 3 Internal Medicine, New York Medical College, Metropolitan Hospital Center, New york, USA

**Keywords:** hospital mortality, vasopressor support, invasive ventilation, septic shock, severity of sepsis, origin of sepsis, apache iv

## Abstract

Objective

The objective of this study is to compare the outcomes of hospital mortality, the requirement of invasive ventilation, vasopressor requirement, duration of vasopressor requirement, and duration of intensive care unit (ICU) stay among the different causes of sepsis and to determine which cause of sepsis had the most severe outcomes.

Methods

A retrospective chart review was done in critically ill adult patients who were admitted with sepsis to the ICU from July 2017 until July 2019. Acute Physiology and Chronic Health Evaluation (APACHE) IV scores were calculated on patients admitted to ICU on day one of ICU admission. Each patient was then evaluated for outcomes of hospital mortality, need for invasive ventilation, requirement of vasopressors, duration of vasopressors, and duration of ICU stay. The outcomes were then compared between the different sources of sepsis to determine which source of sepsis had the highest severity.

Results

In total, 176 patients were included in the study. Ninety-three patients were admitted with respiratory sepsis, 26 patients were admitted with gastrointestinal sepsis, 31 patients were admitted with urosepsis, and 26 patients were admitted with other miscellaneous causes of sepsis. The hospital mortality was highest in the respiratory sepsis group at 32%, with a trend towards statistical significance with a P value of 0.057. ICU stay duration was highest in patients with respiratory sepsis at six days, with a statistically significant P value of < 0.001. The need for invasive ventilation was highest in patients with respiratory sepsis at 64%, with a statistically significant P value of < 0.001. The requirement of vasopressor support was highest in patients with respiratory sepsis at 47% and the duration of vasopressors was highest in both respiratory and gastrointestinal sepsis at three days, however, there was no statistical significance.

Conclusion

Among the different origins of sepsis, the patients with respiratory sepsis had the most severe outcomes, with the highest need for invasive ventilation and the highest ICU stay duration.

## Introduction

Sepsis is a life-threatening condition characterized by organ dysfunction due to an extreme host response to infection, resulting in a complex syndrome of physiologic and biochemical abnormalities [1]. The process of sepsis begins with a focus on infection and the release of toxins that trigger proinflammatory mediators, which causes a widespread and systemic response resulting in tissue injury and organ dysfunction [2]. Sepsis has an incidence estimated at 49 million cases worldwide, associated with 11 million sepsis-related deaths in 2017, representing nearly 20% of all global deaths as per the Global Burden of Disease Study [3]. Mortality of sepsis accounts for more than 50% of hospital deaths, and mortality increases dramatically with greater disease severity: 10-20% for sepsis, 20-40% for severe sepsis, and 40-80% for septic shock [4]. Sepsis is a significant public health concern, ranks highest among admissions for all disease states, and accounts for over $24 billion in hospital expenses, representing 13% of total U.S. hospital costs [5]. There is a rise in the incidence of sepsis secondary to an aging population with multiple comorbidities [6]. It is estimated that sepsis is a leading cause of mortality and critical illness worldwide [7]. Furthermore, the patients who survive sepsis have long-term physical, psychological, and cognitive impacts, leading to significant healthcare implications [8].

There is a need to develop better treatment strategies and understand the heterogeneity in the host response to sepsis [9]. An uncontrolled infection in an organ sets off a dysregulated chain reaction resulting in sepsis [10]. There is limited understanding of the difference in host reaction during sepsis [10]. Clinical studies based on the origin of infection have shown important origin-based variations in patient features, organ dysfunction, cultured micro-organisms, treatment modalities, and duration of hospital stay [10,11]. Mortality from septic shock is primarily determined by a systemic inflammatory response independent of the inciting infection, but the anatomic origin of infection can also play an important role in the outcome of septic shock [11]. Current knowledge indicates that the prognosis, treatment, severity, and time course differ depending on the origin of the infection [12]. 

Acute Physiology and Chronic Health Evaluation (APACHE) IV score is the most recent of the APACHE score series used in predicting hospital mortality in critically ill patients with sepsis. APACHE IV has been found to be superior to APACHE II & III in predicting hospital mortality [13,14]. In this research, we aim to investigate the outcomes of hospital mortality, need for invasive ventilation, requirement of vasopressors, duration of vasopressors and duration of ICU stay in patients admitted with sepsis from different sources of infections using the APACHE IV scoring system. The different origins of sepsis were respiratory, gastrointestinal, urologic, and other miscellaneous causes of sepsis. We hypothesize that outcomes of hospital mortality, need for invasive ventilation, requirement of vasopressors, duration of vasopressors and duration of ICU stay differ between respiratory, gastrointestinal, urologic, and miscellaneous sepsis based on different host response aberrations and that the origin of sepsis plays a significant role in severity and outcome of sepsis. To evaluate the hypothesis, we designed a retrospective observational analysis in a New York City Hospital ICU during a two-year period. By applying the APACHE IV score in determining the outcomes of the patients, we provide a comprehensive observation into the dysregulated host reactions in sepsis with patients classified according to the origin of infection at the time of ICU admission.

## Materials and methods

Study design

A retrospective chart review was done in critically ill adult patients who were admitted with sepsis to the ICU in Metropolitan Hospital from July 2017 until July 2019. Patients were included if they had an ICD-10 discharge code for sepsis, age greater or equal to 18 years at the time of admission, and were admitted to the Metropolitan Hospital Center ICU. APACHE IV scores were calculated on patients admitted to ICU on day one of ICU admission. Each patient was then evaluated during ICU management for outcomes of the hospital mortality, need for invasive ventilation, requirement of vasopressors, duration of vasopressors, and duration of ICU stay. The outcomes were then compared between the different sources of sepsis to determine which source of sepsis had the highest severity. The study was approved by the Brany Institutional Review Board and by the New York City Health and Hospitals Corporation (HHC) central office.

Data collection

Patient data was extracted retrospectively from medical records. The study included a total of 176 patients who were admitted with sepsis in the ICU. Data was collected from electronic medical records. Data collection on ICU admission included demographic data, admission category, source of admission, admission diagnosis, clinical, laboratory data, and chronic health conditions needed to calculate the APACHE IV score. Data collected during the patient stay were the outcomes of hospital mortality, the need for intubation and mechanical ventilation, the need for vasopressors, the duration of vasopressor support in days, and the duration of ICU stay in days.

Data analysis

Descriptive statistics are summarized overall and by causes of sepsis. Frequencies and percentages are reported for categorical variables, mean and standard deviation or medians, and interquartile ranges for continuous measures as appropriate. Differences in demographics and outcomes of hospital mortality, ICU stay, vasopressor requirement, duration of vasopressors among patients on vasopressors, and the need for invasive ventilation across different sources of sepsis were examined using Pearson’s chi-squared test for categorical measures and one-way Analysis of Variance (ANOVA) or Kruskal-Wallis test, as appropriate, for continuous variables. Statistical significance was assessed using a two-sided type I error rate of 0.05. Statistical analyses were conducted using R version 4.1.0 within RStudio version 1.4.1717 (RStudio Team (2020). RStudio: Integrated Development for R. RStudio, PBC, Boston, MA)

## Results

The study included a total of 176 patients who were admitted with sepsis in the ICU. The mean age of the patients was 64 years. There were 95 male patients and 81 female patients (Table 1). Of the 176 patients, 93 patients were admitted with respiratory sepsis, 26 patients were admitted with gastrointestinal (GI) sepsis, 31 patients were admitted with urosepsis, and 26 patients were admitted with other miscellaneous causes of sepsis, which included cutaneous sepsis, osteomyelitis, dental abscess, epidural abscess, permacath associated sepsis and undifferentiated sepsis (Table 1). Community-acquired pneumonia was the leading diagnosis of respiratory sepsis. Bowel perforation and gastroenteritis were the leading causes of gastrointestinal sepsis. Pyelonephritis was the leading cause of urosepsis. Cellulitis and post-amputation leg sepsis were the leading causes of miscellaneous sepsis (Table 2). Table 3 summarizes the results of the study.

**Table 1:** Demographics and Subgroups of Sepsis

Total number of patients	N = 176
Age	64 (17.5)^2^
Sex	
Male	95 (54.0%)
Female	81 (46.0%)
Causes of Sepsis	
Respiratory Sepsis	93 (52.8%)
GI Sepsis	26 (14.8%)
Urosepsis	31 (17.6%)
Miscellaneous^1^	26 (14.8%)
^1^Miscellaneous categories of sepsis include osteomyelitis, cutaneous sepsis, dental abscess, epidural abscess, invasive lines, undifferentiated sepsis	
^2^Mean (SD); n (%)	

**Table 2:** Causes of Sepsis

	N = 176
Respiratory Sepsis	93
Community Acquired Pneumonia	93 (100.0%)
GI Sepsis	26
Acute Cholecystitis	2 (7.7%)
Bowel Obstruction	2 (7.7%)
Bowel Perforation	9 (34.6%)
Colitis	1 (3.8%)
Gastroenteritis	9 (34.6%)
Pancreatitis	1 (3.8%)
Retroperitoneal Abscess	1 (3.8%)
Spontaneous Bacterial Peritonitis	1 (3.8%)
Urosepsis	31
Pyelonephritis	30 (96.8%)
Vesicovaginal Fistula	1 (3.2%)
Miscellaneous Sepsis	26
Cellulitis	5 (19.2%)
Dental Abscess	1 (3.8%)
Epidural Abscess	1 (3.8%)
Gangrene	1 (3.8%)
Osteomyelitis	2 (7.7%)
Permacath Line Sepsis	1 (3.8%)
Post Leg Amputation Sepsis	5 (19.2%)
Septic Arthritis	1 (3.8%)
Skin Abscess	1 (3.8%)
Undifferentiated Sepsis	8 (30.8%)

**Table 3:** Differences in Characteristics and Outcomes by Subgroups of Sepsis MICU: Medical Intensive Care Unit

	Overall, N = 176^2^	Respiratory Sepsis, N = 93 (53%)^2^	GI Sepsis, N = 26 (15%)^2^	Urosepsis, N = 31 (18%)^2^	Miscellaneous^1^, N = 26 (15%)^2^	p-value^3^
Age	64.0 (17.5)	62.2 (17.0)	67.2 (17.1)	64.7 (19.9)	66.2 (17.1)	0.507
Sex						0.757
Male	95 (54.0%)	48 (51.6%)	16 (61.5%)	18 (58.1%)	13 (50.0%)	
Female	81 (46.0%)	45 (48.4%)	10 (38.5%)	13 (41.9%)	13 (50.0%)	
Outcomes						
In-Hospital Mortality						0.057
No	133 (75.6%)	63 (67.7%)	21 (80.8%)	28 (90.3%)	21 (80.8%)	
Yes	43 (24.4%)	30 (32.3%)	5 (19.2%)	3 (9.7%)	5 (19.2%)	
MICU Stay (days)	4.0 (3.0, 9.2)	6.0 (3.0, 11.0)	4.0 (2.2, 6.8)	3.0 (2.0, 5.0)	3.0 (2.0, 6.0)	<0.001
Invasive Ventilation						<0.001
No	96 (54.5%)	33 (35.5%)	16 (61.5%)	25 (80.6%)	22 (84.6%)	
Yes	80 (45.5%)	60 (64.5%)	10 (38.5%)	6 (19.4%)	4 (15.4%)	
Vasopressor						0.132
No	107 (60.8%)	49 (52.7%)	19 (73.1%)	21 (67.7%)	18 (69.2%)	
Yes	69 (39.2%)	44 (47.3%)	7 (26.9%)	10 (32.3%)	8 (30.8%)	
Patients on Vasopressors						
Duration of Vasopressor (days)	3.0 (1.0, 5.0)	3.0 (1.0, 5.0)	3.0 (1.5, 4.5)	1.0 (1.0, 1.8)	2.5 (1.0, 3.5)	0.128
^1^Miscellaneous sepsis categories include Osteomyelitis, Cutaneous, Dental Abscess, Epidural Abscess, Invasive Lines, Unknown Origin						
^2^n (%); Mean (SD); Median (IQR)						
^3^Pearson's Chi-squared test; One-way ANOVA; Kruskal-Wallis test						

**Figure 1:**
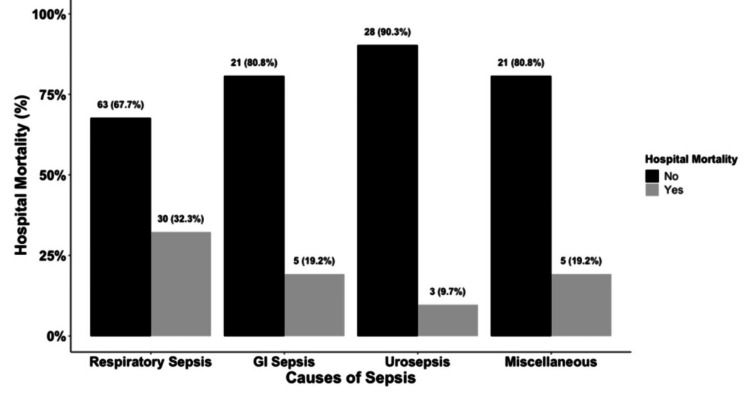
Bar Graph of Hospital Mortality among Subgroups of Sepsis

**Figure 2:**
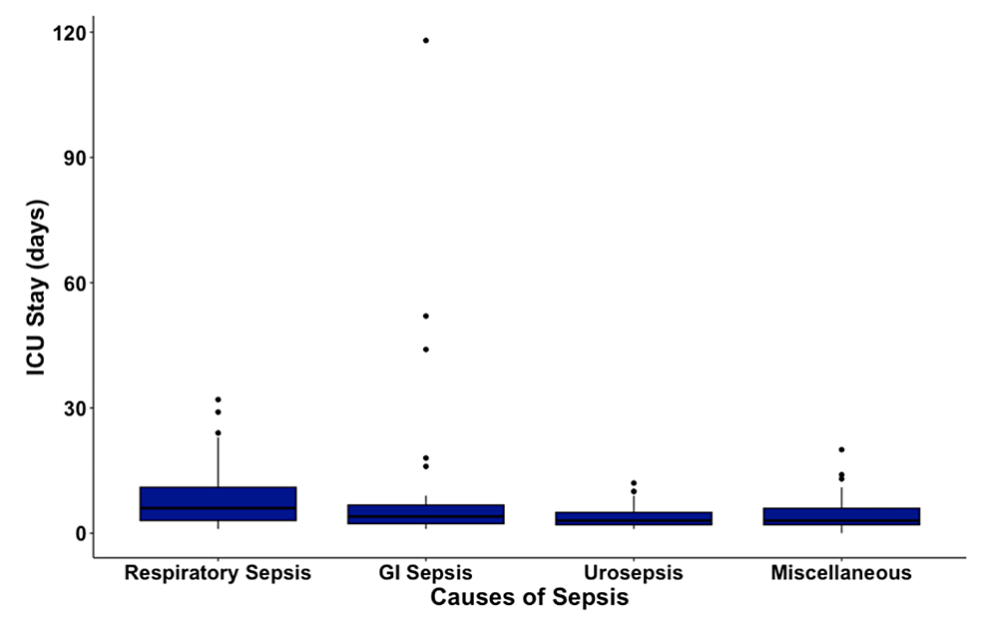
Box Plot of ICU stay (days) among Subgroups of Sepsis

The hospital mortality was highest in the respiratory sepsis group at 32%, followed by gastrointestinal sepsis and sepsis from miscellaneous causes at 19%, with a trend towards statistical significance with a P value of 0.057 (Figure 1). ICU stay duration was highest in patients with respiratory sepsis at six days, followed by gastrointestinal sepsis at four days, followed by urosepsis and miscellaneous sepsis at three days, with a statistically significant P value of < 0.001 (Figure 2). 

**Figure 3:**
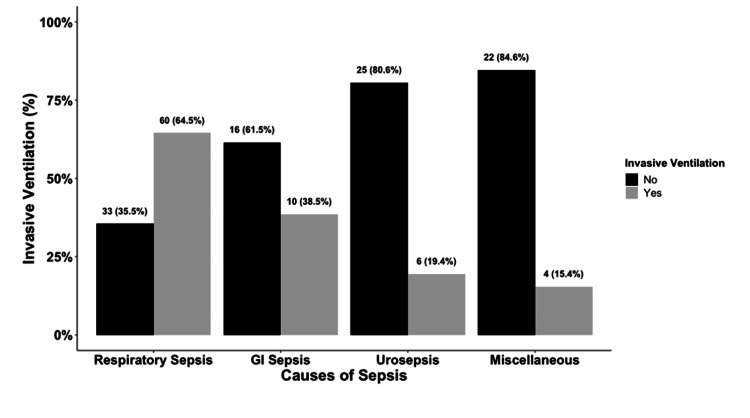
Bar Graph of Invasive Ventilation among Subgroups of Sepsis

**Figure 4:**
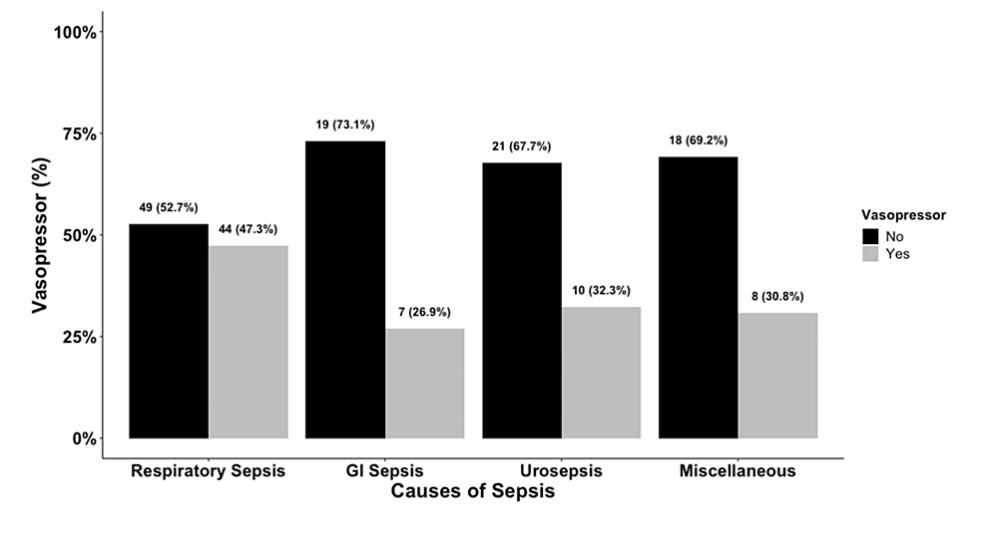
Bar Graph of Vasopressor Requirement among Subgroups of Sepsis

**Figure 5:**
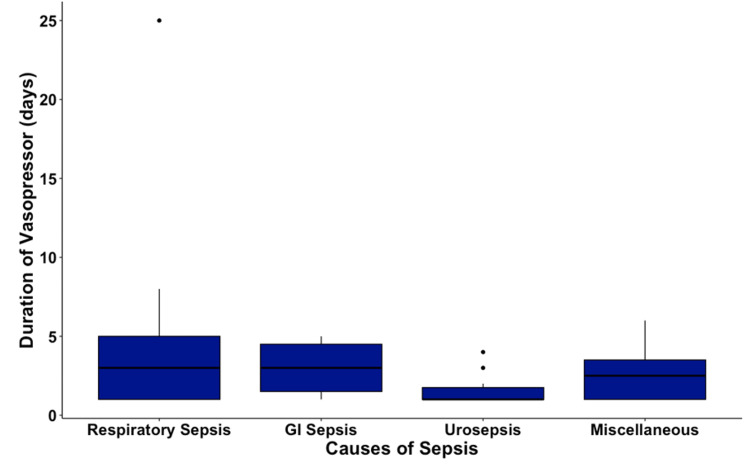
Box Plot of Duration of Vasopressor (days) among Subgroups of Sepsis

The need for invasive ventilation was highest in patients with respiratory sepsis at 64%, followed by gastrointestinal sepsis at 38%, with a statistically significant P value of < 0.001 (Figure 3). The development of septic shock leading to the requirement of vasopressor support was highest in patients with respiratory sepsis at 47%, followed by patients with urosepsis at 32%; however, there was no statistical significance with a P value of 0.132 (Figure 4). The duration of vasopressors was highest in both respiratory and gastrointestinal sepsis at three days, and there was no statistical significance with a P value of 0.128 (Figure 5).

## Discussion

This study illustrates that the origin of infection leading to sepsis is independently associated with the severity of sepsis, as indicated in previous studies [2,11,15-17]. Patients who had respiratory sepsis had a greater need for intubation and mechanical ventilation and longer ICU stay compared with patients having gastrointestinal, urosepsis, and miscellaneous causes of sepsis. Hospital mortality, the requirement for vasopressor support, and the duration of vasopressor support were also highest in patients with respiratory sepsis, although they were not statistically significant. Patients with gastrointestinal sepsis had the next most severe presentation of sepsis after patients with respiratory sepsis based on the outcomes of invasive ventilation, duration of ICU stay, hospital mortality, and vasopressor requirement. 

In our study, the highest mortality was noted for patients with respiratory sepsis, followed by gastrointestinal sepsis, sepsis from miscellaneous causes, and urosepsis in the order of decreasing mortality. The hospital mortality showed a trend toward statistical significance with a p-value of 0.057. The origin-specific severity reported in this study is in accordance with previous clinical studies, which found markedly higher mortality in patients with respiratory sepsis followed by the gastrointestinal tract, whereas sepsis from the genitourinary tract had lower mortality, indicating the origin of infection could be an independent predictor of mortality and severity of sepsis [2,11,15-17].

Sepsis and septic shock are primarily driven by a profound proinflammatory state, which initially contributes to eradicating invading organisms but subsequently leads to an immunocompromised state and tissue injury [18,19]. Our data suggest that the anatomic origin of infection significantly influences the outcome of sepsis [11]. In our study, it is notable that the most severe outcomes of highest hospital mortality, highest need for intubation, highest ICU duration stay, and highest vasopressor requirement are associated with infections with large burdens of organisms such as in respiratory sepsis. This large burden of organisms and antigens contributes to the state of systemic immune exhaustion and profound immunosuppression, irrespective of predisposing factors, comorbid conditions, and treatment strategies [18,20]. The differences noted in the outcomes of the study, based on the origin of sepsis, are likely secondary to protective barriers and natural defense mechanisms unique to each organ [2]. Certain infections, such as obstructive uropathy-associated urinary tract infections, are more promptly recognized, and rapid source control by relieving obstruction may contribute to their least severity and lower mortality [11].

In our study, patients hospitalized with respiratory sepsis had an average ICU stay of six days, followed by gastrointestinal sepsis at four days and urosepsis and miscellaneous sepsis at three days each. We noticed a trend that the length of stay in the ICU varies significantly based on the origin of sepsis [21]. Understanding these differences is crucial for healthcare organizations for better utilization of resources since longer ICU duration of stay directly increases the cost expenditure and could also help physicians provide more definite information to families regarding patients' expected length of stay and financial costs and expenditure [2].

Earlier studies have focused on the immunologic component of sepsis due to the concept that the inflammatory cascade, once triggered, progresses independently of the infectious trigger [22,23]. However, due to this fundamental misconception, several attempts at immunomodulatory therapies have failed to show improved outcomes in clinical trials [20]. The drawback of this immunologic concept is the failure to acknowledge the gravity of the infectious process and the role of microbial load in driving the pathogenesis leading to organ dysfunction [2]. The origin of infection is an independent predictor of the severity of sepsis, as determined in this study [2,11,15]. 

The management of sepsis and septic shock has been transformed by landmark clinical trials that emphasized the significance of early, goal-directed resuscitation and antimicrobial therapy [24,25]. Since the introduction of goal-directed guidelines for the management of sepsis and septic shock, there has been a significant decline in mortality from sepsis and septic shock [6,26]. At the same time, several clinical trials based on treatment for sepsis and septic shock have not demonstrated any significant differences between intervention and control groups [11,27-29]. This might be primarily because of the heterogeneity of patients enrolled in these trials [10]. In an overwhelming number of patients, it has been noted that sepsis has a distinct origin, and the underlying reason for the heterogeneity of sepsis has been regarding differences in the origin of sepsis [10]. Clinical trials in sepsis that analyzed strategies influencing the host reaction largely did not consider the origin of sepsis [10]. Understanding the significant role in the heterogeneity of treatment effect and the role of the origin of infection is important in the design of future clinical trials in more homogeneous groups of patients [11,30]. Our results suggest that the origin of infection partly explains sepsis heterogeneity and should be considered when selecting patients for clinical trials testing the role of immunomodulatory agents such as steroids, monoclonal antibodies, interferon-gamma, and granulocyte-macrophage colony-stimulating factor in the outcomes of sepsis [10]. The results of this study show that the origin of infection constitutes an independent predictor of the severity of sepsis and the host response [10].

There were several limitations in this study. The study had a moderate sample size and did not have a large sample size. The study was a retrospective observational analysis and not a multicenter randomized control trial. The study was conducted in a single center New York City Health and Hospital Corporation facility and caution must be used in generalizing the results of this study to other clinical settings. Out-of-hospital mortality and one-year mortality post-discharge were not considered in the study and the time frame was only up to the discharge of the patient from the hospital. 

## Conclusions

In our study, among the different origins of sepsis, the patients with respiratory sepsis had the highest severity. This study confirms that the origin of infection is an independent predictor of the severity of sepsis. The outcome of the study further explains the heterogeneity of sepsis and suggests that the origin of infection partly contributes to the heterogeneity of sepsis. Our study further indicates in the future, the origin of infection should be considered when identifying patients for clinical trials evaluating immunomodulatory agents for patients with sepsis and septic shock.
